# Radiotherapy waiting times for women with breast cancer: a population-based cohort study

**DOI:** 10.1186/1471-2407-7-71

**Published:** 2007-05-01

**Authors:** Ruth H Jack, Elizabeth A Davies, David Robinson, Richard Sainsbury, Henrik Møller

**Affiliations:** 1 King's College London, Thames Cancer Registry, 1^st ^Floor Capital House, 42 Weston Street, London, SE1 3QD, UK; 2 University College London, Department of Surgery, The Medical School Building, 74 Huntley Street, London, WC1E 6AU, UK

## Abstract

**Background:**

Waiting times for cancer patients are a national priority in the UK. Previous studies have shown variation between cancer networks in the time between diagnosis and start of radiotherapy for all cancer patients. Studies of the relationship between delay in receiving treatment and survival of breast cancer patients have been inconsistent. This study aimed to examine factors associated with waiting times for radiotherapy for breast cancer patients.

**Methods:**

35,354 women resident in South East England and diagnosed with breast cancer between 1992 and 2001 who received radiotherapy within six months of diagnosis were identified from the Thames Cancer Registry. Time to radiotherapy was measured from either the date of diagnosis or the start of the previous treatment, whichever was shorter. Unadjusted and adjusted logistic regression models were fitted to examine whether patients received radiotherapy within 60 days of their diagnosis or previous treatment.

**Results:**

The adjusted proportions of patients receiving radiotherapy within 60 days varied significantly between different cancer networks (range: 43% to 81%), and decreased from 68% in 1992 to 33% in 2001. After adjustment there was no association between deprivation of area of residence, age or stage and radiotherapy wait. Median time waited to radiotherapy increased over the study period whether measured from the start of chemotherapy, hormone therapy, surgery or the date of diagnosis.

**Conclusion:**

This study covered a period of time before the investment following the Cancer Plan of 2000. Results are consistent with other findings suggesting variation between cancer networks and increasing waits over time. Further studies should examine different methods of measuring waiting time, the causes and consequences of waits for radiotherapy and the effect of current initiatives and investments.

## Background

In recent years the National Health Service in England and Wales has focused attention and considerable resources on reducing waiting times for cancer patients. In 2000 a large survey [1] based on cases diagnosed in 1997 found that the waiting times of patients with cancer in England varied across regions. The shortest waits were found for patients with breast cancer, who waited a median of 14 days from referral to their first outpatient appointment and 35 days to first definitive treatment. This group was the first for whom a maximum two-week wait between urgent referral and first appointment at hospital was proposed [2]. Implementation began in 1999 and the national cancer waiting times database now shows that nearly all urgently referred breast cancer patients are seen within two weeks [3]. As this first target has been met, attention has turned to the wait between diagnosis and treatment. A further target of a maximum one-month wait from diagnosis to treatment was also met for 99.7% of patients in the last quarter of 2005/2006, and a one-month wait from urgent referral to beginning of treatment for all cancers has been proposed for the year 2008 [3,4].

The importance of reducing the length of time between first symptoms and treatment of breast cancer was strongly suggested by a systematic review by Richards *et al *[5]. These authors reviewed 87 studies of breast cancer, including data on over 100,000 patients. They found that those patients who had waited three months or more from first symptoms to treatment had 12% lower survival than those waiting shorter periods. However, the patients with the longer delays were more likely to have advanced stage of disease by the time they reached treatment. Once the effect of stage was taken into account, delays were not associated with reduced survival. A study of patients in Yorkshire demonstrated that breast cancer patients waiting more than 30 days between family physician referral and treatment had better survival than those waiting 30 days or less [6].

The relationship between delay in diagnosis and treatment (whether attributable to the patient, the doctor or the health care system), and survival from breast cancer is complex. At each point from initial symptoms to definitive treatment, the decisions and actions of patients and doctors may be influenced by their assessment of the severity of symptoms, the treatment needed and the services available. For example, one Danish study found that patients for whom the delay attributable to the doctor had been longer had a better prognosis than those for whom it had been shorter. This suggested that doctors identified patients with more aggressive breast tumours and sent them through the system more quickly [7]. Similarly, one German study found that the relationship between stage of disease at diagnosis and delay before treatment showed a U-shaped association. Women with either very short or very long delays had the highest proportions of late-stage tumours. This suggested that women presenting with a clear clinical picture of later stage disease may have been diagnosed and treated more quickly, while those presenting with very early symptoms (and a less clear clinical picture) may have initially received a delayed diagnosis and therefore reached treatment with more advanced disease [8]. In a study of women with breast cancer in South East England, Robinson *et al *[9] found that the length of wait from first hospital appointment to first treatment was highly dependent on what that first treatment was. The group for whom this was radiotherapy experienced the longest delays, with just over half receiving treatment within five weeks of first hospital appointment. A recent study by Mikeljevic *et al *[10] found that patients treated with conservative surgery had a higher mortality if they waited longer than nine weeks for subsequent radiotherapy.

In a study of waiting times for radiotherapy in patients diagnosed with cancer between 1992 and 2001 in South East England we found variation across cancer networks and different cancer sites [11]. The difference in waiting times between networks persisted after adjustment for age, sex, cancer site, socioeconomic deprivation and year of diagnosis, with the proportion receiving radiotherapy within 60 days of diagnosis ranging from 44% to 71%. Repeating the analysis using a 'truncated' waiting time (that from diagnosis or the last treatment received before radiotherapy), we found generally shorter waits but a similar pattern of variation. The study also revealed a significant increase in waiting times between 1992 and 2001. This increase is consistent with a recent report from the Royal College of Radiologists which found that between 1998 and 2002, towards the end of our study period, the proportion of patients waiting longer than four weeks to start potentially curative radical radiotherapy had increased from 28% to 81% across the UK [12].

This paper concentrates on patients diagnosed with breast cancer in South East England between 1992 and 2001 who received radiotherapy within six months of diagnosis. Our aim was to examine in more detail the factors influencing whether patients with breast cancer received radiotherapy within 60 days of their diagnosis or previous treatment.

## Methods

### Data

In the United Kingdom, cancer registries record the occurrence of cancer in their resident populations as well as treatments given in the first six months after diagnosis. Registration is initiated by clinical and pathology information received from hospitals and by information about deaths provided by the National Health Service Central Register through the Office for National Statistics. Data collection officers collect further information on demographic details, disease stage and treatment from the medical records. Data are quality assured as they are added to the central database. The date of diagnosis is defined as the date on which cancer was confirmed by the most precise of the diagnostic tests performed (ideally histological or cytological confirmation) or, if not available, the date of admission to hospital for the malignancy or, if there was no admission, the date of first outpatient consultation. During the period of study the Thames Cancer Registry covered the population of an area of South East England covering Essex, Hertfordshire, London, Kent, Surrey and Sussex.

Records on female patients registered with breast cancer (invasive or in situ) at Thames Cancer Registry who were diagnosed between 1992 and 2001 were examined. Of the 95,118 eligible patients, 35,354 (37%) received radiotherapy within six months of diagnosis, and this subgroup was used in all further analyses. A relatively small number of patients (n = 725, 0.8%) were excluded from the study at the outset either because the date recorded for their diagnosis appeared to be after their radiotherapy or after their death, or because their recorded date of radiotherapy was after their death.

Age was divided into ten-year groups. The staging data available to the registry relies on that recorded by clinicians within the medical records. Because this is often not completely recorded, the registry uses all the information that it can obtain to classify stage using a simplified system based on the WHO Extension of Disease Classification. This "TCR staging" is: 1) 'localised'; 2) 'extension beyond the organ of origin'; 3) 'local lymph node involvement'; 4) 'metastases'; or 'not known'. Postcode of residence at diagnosis was used to determine cancer network and ward of residence. Socioeconomic deprivation of the patient's area of residence was estimated by a ward level quintile of the income score part of the Indices of Multiple Deprivation (IMD) [13].

### Analysis

This study follows similar methodology to that used in the previous analysis of all cancer patients [11]. Time to radiotherapy was measured from either the date of diagnosis or the start of the most recent previous treatment, whichever was shorter. If a patient's first treatment was radiotherapy we measured the time they waited to radiotherapy from the date of their diagnosis. For patients who received another treatment first, we used the start date of the treatment they received immediately before radiotherapy. This definition was used as radiotherapy tends to be given after other therapies, and the waiting time from date of diagnosis only may be exaggerated by waits for other treatments. While it would be preferable to measure time waited to radiotherapy starting at the date other treatments were completed, particularly chemotherapy [14], unfortunately this information is not available in the Thames Cancer Registry dataset.

The median waiting time between diagnosis or previous treatment and the start of radiotherapy was calculated, stratified by whether this was measured from start of chemotherapy, hormone therapy, surgery or the date of diagnosis (if radiotherapy was the first treatment received). Logistic regression models were fitted to examine which factors affected whether the patient received radiotherapy within 60 days of diagnosis or previous treatment. A fully adjusted model was fitted, including cancer network of residence, year of diagnosis, site (invasive or in situ), stage, IMD income quintile, age group, and a variable indicating whether the patient survived six months after diagnosis. The latter was included to allow for the fact that short-term survivors who had been given radiotherapy would of necessity receive it soon after diagnosis. All results from logistic regression modelling were transformed to give crude and adjusted proportions receiving radiotherapy within 60 days of diagnosis or previous treatment. The 60-day target was chosen to be consistent with our previous work [11] and, as around 60% of patients met the target, this cut-off point provided adequate statistical power for our analysis. Tests for trend were done by fitting categorical variables as continuous, and chi^2 ^tests were used to test for heterogeneity, excluding other and not known categories.

## Results

**Figure 1:**
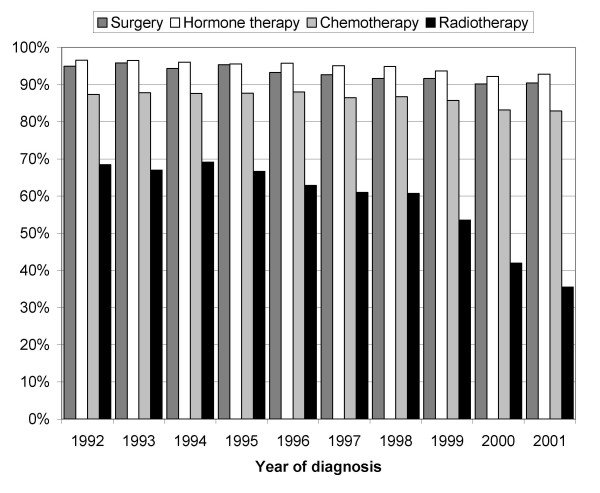
Proportion of patients receiving different types of treatment within 60 days of diagnosis or previous treatment by year of diagnosis

Figure 1 shows the waiting times from diagnosis or previous treatment for the main types of treatment received by patients with breast cancer. The waiting times for surgery, hormone therapy and chemotherapy were short and more than 80% of patients waited less than 60 days for these treatments. There was little change in these waits over the study period. The waiting times for radiotherapy, however, were longer and the proportion of patients that received radiotherapy within 60 days decreased from more than 60% in the early 1990s to less than 40% in 2001. The variation in waiting time for radiotherapy is the subject of all subsequent analyses.

**Figure 2:**
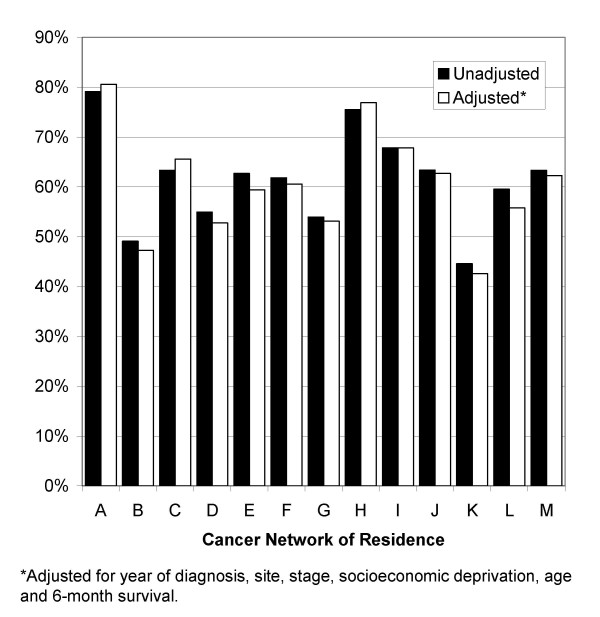
Proportion of patients receiving radiotherapy within 60 days of diagnosis or previous treatment by cancer network of residence

The proportions of patients receiving radiotherapy within 60 days of their diagnosis or previous treatment are shown in Table 1, both unadjusted and adjusted for all other variables. There was a highly significant variation in the proportions in relation to cancer network of residence. Between 45% and 79% of patients were treated within 60 days. These proportions corresponded to median waits ranging from 36 to 65 days in different networks. Statistical adjustment had no material influence on this variation between cancer networks (Figure 2).

**Figure 3:**
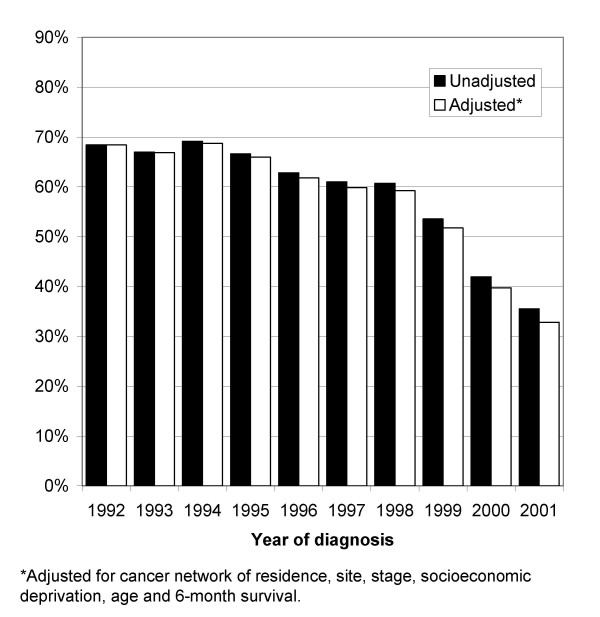
Proportion of patients receiving radiotherapy within 60 days of diagnosis or previous treatment by year of diagnosis

There was an increase in median wait for radiotherapy over time from 43 days in 1992 to 77 days in 2001. This increase was also reflected in the significant decrease in the proportions of patients receiving radiotherapy within 60 days (from 68% in 1992 to 36% in 2001). This variation in waiting times over the study period was not materially influenced by statistical adjustments (Figure 3).

**Figure 4:**
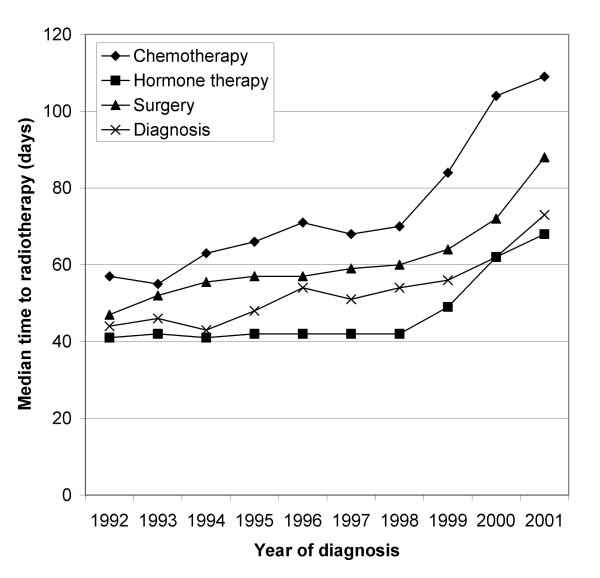
Median time to radiotherapy (days) by year of diagnosis, whether measured from start of chemotherapy, hormone therapy, surgery or date of diagnosis

Figure 4 shows the median time waited to radiotherapy, whether this was measured from the start of chemotherapy, hormone therapy, surgery or the date of diagnosis. Although patients who received chemotherapy immediately before radiotherapy consistently had the longest waits, the time to waited for radiotherapy increased in the later period in all groups.

Patients with invasive cancer were significantly more likely to receive radiotherapy within 60 days (60%) than those with carcinoma in situ (50%) (p < 0.0001). This difference was again unchanged after adjustment for other variables. The association between stage of disease and wait for radiotherapy was more complex. The unadjusted proportions indicated that patients with metastases were more likely to receive radiotherapy within 60 days than other patients. However, after adjusting for other variables this difference became statistically non-significant. The attenuation of this stage effect in the adjusted analysis was mainly due to adjustment for survival, which is very closely associated with stage. There were no differences in waiting time between socioeconomic groups or between age groups.

## Discussion

This study of breast cancer patients treated with radiotherapy in South East England found an increase in the time they had waited after their diagnosis or previous treatment before receiving radiotherapy over the period 1992 to 2001. Considerable variation in waiting times for radiotherapy between cancer networks also emerged. Both findings were robust to adjustment for case mix factors including age, stage and deprivation of area of residence.

There are a number of factors to consider in interpreting the results of this study, some of which we were not able to measure. Firstly, increasing waits could be partially explained by a heavier overall workload or increasing complexity of treatment in radiotherapy departments over time. We could only include patients in this study if they received radiotherapy within six months of diagnosis, as this is the full follow-up period for collection of treatment information by the registry. Radiotherapy received after this period would not have been recorded in the database, but would still contribute to an increased workload in radiotherapy departments within networks. Patients included in this study will not be representative of all patients receiving radiotherapy, but relate to patients receiving radiotherapy as part of their initial treatment. Although the numbers of all cancer patients being treated with radiotherapy within six months of diagnosis was fairly constant for South East England [11], treatment is likely to have become more complex over the study period and require more detailed planning. For example, a study by the Royal College of Radiologists [15] found that between 1992 and 1997 the number of new patients had increased by 8.6%, whereas the number of exposures had risen by 18%.

Secondly, we attempted to assess the "true" wait for radiotherapy for patients who had received other treatments before radiotherapy by measuring their waiting time from the start of the last treatment before radiotherapy. However, this approach does not overcome all problems. Only the start date of the previous treatment was available, so the duration of that treatment and of any recovery time were included within the wait. This will have affected the wait from chemotherapy to radiotherapy in particular, as chemotherapy can be a lengthy treatment. However, we found that the increase in wait over time was seen whether the previous treatment was chemotherapy, hormone therapy or surgery as well as when radiotherapy was itself the first treatment received after diagnosis. The time taken by further investigations after diagnosis and before treatment will also be included in the wait measured here. Increasing waits for these investigations could in turn be contributing to longer waits for radiotherapy.

Thirdly, while adjustment for several important variables did not explain the variation over time or between networks it may have been incomplete, particularly for stage which was not known for 24% of patients receiving radiotherapy. Other factors which would have an important influence on time waited to radiotherapy, such as comorbidity, are not available in the Thames Cancer Registry dataset and could not be included in this study. Those with metastatic disease would receive palliative, rather than curative, radiotherapy, and would therefore be treated more quickly. The fact that patients with the less severe breast cancer in situ waited longer for radiotherapy highlights the fact that the observed differences will often be a consequence of clinical decisions.

It is a weakness of the cancer registration data that only treatment episodes within the six month period following diagnosis are routinely recorded. From the incomplete records we have of later treatments, it is evident that an increasing proportion of breast cancer patients received radiotherapy later than six months after diagnosis. The effect of this artefact is conservative with respect to our finding of a trend towards longer waits for radiotherapy, but it also implies that the women who received radiotherapy seven months after diagnosis or later were entirely missed in the analysis. As the time to treatment increases beyond six months, it becomes more difficult to decide whether the radiotherapy was a primary or a secondary treatment choice. In the future it is hoped that cancer registries will include not only treatments and their dates but also treatment decisions and their dates, as this would facilitate waiting times analysis, particularly when the waits are long. Studies which break down the overall time between disease detection and adjuvant treatment into distinct stages are able to identify where intervals are increasing over time [16].

Our findings can be compared to several recent studies which have examined waiting times for radiotherapy. A UK national audit of radiotherapy waiting times for all cancer patients showed an increase in the proportion of patients waiting longer than the Joint Collegiate Council for Oncology maximum acceptable standard for radical, palliative and adjuvant treatments between 1998 and 2003 [17]. In our earlier study of all cancer patients for the period 1992 and 2001 we found a similar trend of increasing waits for all cancers [11]. Studies that have looked specifically at breast cancer have also found increasing waiting times over time. For example, time from diagnosis to radiotherapy increased for breast cancer patients in Canada between 1992 and 2000 [18], and Mikeljevic *et al *[10] showed that the wait from breast-conserving surgery to radiotherapy in Yorkshire, UK had increased between 1986 and 1998.

Variation between cancer networks was previously seen for all cancer sites in South East England [11]. The UK study in Yorkshire also found variation between hospital trusts in the time from breast-conserving surgery to radiotherapy for patients who did not have chemotherapy [10]. This again confirmed findings from an earlier study in Yorkshire showing variation in treatment between districts [19].

Studies of the relationship between delay in receiving treatment and survival have been inconsistent [20]. One systematic review found that a longer interval between the onset of symptoms and treatment was linked to worse survival [5]. However when studies restricted the period of waiting to periods involving health services, for example time between family doctor referral and treatment [6] or date first seen to diagnosis [5], better survival was found in patients with longer waits.

Advanced booking of radiotherapy, for example at the time of the multi-disciplinary team meeting, may reduce time spent waiting for treatment. Patient numbers and length of treatment should be monitored and forecast to ensure staff and equipment levels are adequate.

The UK Cancer Plan was launched in 2000 [21], and recognised very clearly that substantial improvement was needed in the provision of radiotherapy services. It is unlikely that the investment that followed this plan would have begun to have an effect in the years 2000 and 2001 included in the last part of our study period. However, as some observers have raised concerns that this investment might not be sufficient [22], close monitoring of the situation will be needed. Future studies should seek to measure the waits for radiotherapy after referral more directly, compare these to those that can be calculated from registry records and understand their causes and possible consequences. One approach to understanding the apparent variation in waiting times between cancer networks would be to compare the workload (number of patients referred and treated) and capacity (use of staff and equipment) of different trusts to waiting times for patients. Such work may be most efficiently carried out by detailed case studies of several trusts or networks that appear to have widely differing waits. The effect of cancer networks on waiting times for radiotherapy should be explored further, for example by looking at variation within networks, the effect of the size of population covered or treated, or distance to the radiotherapy suite. The possible influence of these waiting times on survival for patients with breast cancer of different stage will need to be explored in detail and reviewed as current policy initiatives begin to take effect.

Future studies should explore the effect of different treatment choices on waiting times for radiotherapy, for example type of surgery and whether chemotherapy was received or not, as the role of radiotherapy is influenced by these factors. In addition, the effect of treatment delays on outcomes (particularly survival) should be examined in detail.

## Conclusion

The findings of increasing time to radiotherapy for women with breast cancer in South East England and variation in time waited between cancer networks suggests that better planning and more investment in radiotherapy services are needed. Further studies should explore the association between waiting time and survival and the psychological impact of long waits for radiotherapy.

## Competing interests

The author(s) declare that they have no competing interests.

## Authors' contributions

RHJ was involved in the conception and design of the study, performed the analysis, interpreted the data and helped to draft the manuscript.

EAD was involved in the conception and design of the study, interpreted the data and helped to draft the manuscript.

DR was involved in the conception and design of the study, performed some of the analysis, interpreted the data and revised the manuscript.

RS interpreted the data and revised the manuscript.

HM was involved in the conception and design of the study, interpreted the data and revised the manuscript.

All authors read and approved the final manuscript.

## Pre-publication history

The pre-publication history for this paper can be accessed here:



## Figures and Tables

**Table 1:** Median waiting times and proportion of patients receiving radiotherapy (RT) within 60 days of diagnosis or previous treatment

	Total number of patients	Number(%) receiving RT	Median wait (days)	% treated within 60 days after diagnosis or previous treatment	
				Unadjusted	Adjusted^a^

**Network of residence**					
A^b^	1940	489 (25)	36	79	81
B	11527	4685 (41)	61	49	47
C^b^	4626	1716 (37)	48	63	66
D^b^	7239	3190 (44)	56	55	53
E	8154	2924 (36)	49	63	59
F	7196	2665 (37)	49	62	61
G	8768	2976 (34)	56	54	53
H	5171	1806 (35)	42	76	77
I*	8915	2977 (33)	44	68	68
J	9793	3572 (36)	48	63	63
K	9396	3000 (32)	65	45	43
L^b^	2111	650 (31)	55	60	56
M	10197	4677 (46)	48	63	62
Other/Not known	85	27 (32)	55	63	64
Test for heterogeneity:			χ^2 ^(12 df)	*958.9*	*1046.7*
				*p < 0.0001*	*p < 0.0001*
**Year of diagnosis**					
1992*	9163	3555 (39)	43	68	68
1993	8403	3099 (37)	45	67	67
1994	8911	3530 (40)	45	69	69
1995	9228	3718 (40)	47	67	66
1996	9937	3935 (40)	49	63	62
1997	10362	4134 (40)	51	61	60
1998	10011	3672 (37)	51	61	59
1999	9944	3667 (37)	56	54	52
2000	9693	3127 (32)	69	42	40
2001	9466	2917 (31)	77	36	33
Test for trend:			χ^2 ^(1 df)	*1229.7*	*1329.0*
				*p < 0.0001*	*p < 0.0001*
**Site**					
Breast*	89163	34162 (38)	51	60	60
Breast in situ	5955	1192 (20)	60	50	50
Test for heterogeneity:			χ^2 ^(1 df)	*42.2*	*37.7*
				*p < 0.0001*	*p < 0.0001*
**Stage**					
1*	38870	16969 (44)	54	58	58
2	1955	747 (38)	51	58	53
3	17201	7568 (44)	55	56	54
4	4947	1661 (34)	31	73	68
Not known	32145	8409 (26)	47	63	59
Test for trend:			χ^2 ^(1 df)	*20.8*	*0.8*
				*p < 0.0001*	*p = 0.3660*
**IMD income quintile**					
1 (Least deprived)*	19996	7854 (39)	50	61	61
2	20010	7600 (38)	51	59	59
3	19058	7211 (38)	53	58	59
4	19175	6914 (36)	52	59	59
5 (Most deprived)	16759	5756 (34)	51	59	59
Not known	120	19 (16)	55	58	62
Test for trend:			χ^2 ^(1 df)	*1.8*	*1.6*
				*p = 0.1783*	*p = 0.2050*
**Age group**					
< 50*	19883	8329 (42)	51	59	59
50–59	23091	10483 (45)	50	60	62
60–69	19463	9170 (47)	53	60	62
70–79	17581	5882 (33)	53	58	60
80+	15100	1490 (10)	52	58	59
Test for trend:			χ^2 ^(1 df)	*0.3*	*1.4*
				*p = 0.5828*	*p = 0.2456*
**All cases**	95118	35354 (37)	52	59	

a = Adjusted for all other variables in table and survival

b = TCR part of network

* = Baseline category

Tests for heterogeneity and trend exclude 'other' and 'not known' categories
